# Role of α-Defensin and the Microbiome in Prosthetic Joint Infection: A Prospective Cohort Study in Korea

**DOI:** 10.3390/jcm12185964

**Published:** 2023-09-14

**Authors:** Yae Jee Baek, Youn-Jung Lee, Jung Ah Lee, Jung Ho Kim, Hyuck Min Kwon, Joon-Sup Yeom, Kwan Kyu Park, Su Jin Jeong

**Affiliations:** 1 Division of Infectious Diseases, Department of Internal Medicine, Soonchunhyang University Seoul Hospital, Soonchunhyang University College of Medicine, Seoul 04401, Republic of Korea; stellangela@schmc.ac.kr; 2 Division of Infectious Diseases, Department of Internal Medicine, Severance Hospital, Yonsei University College of Medicine, Seoul 03722, Republic of Korea; 3 Department of Orthopedic Surgery, Severance Hospital, Yonsei University College of Medicine, Seoul 03722, Republic of Korea

**Keywords:** prosthetic joint infection, α-defensin, leukocyte esterase, diagnostic accuracy, microbiome

## Abstract

The utility of α-defensin (AD), leukocyte esterase (LE) levels, and metagenomics sequencing as diagnostic tools for prosthetic joint infection (PJI) has been suggested, but there are few studies among the Asian population. This study aimed to evaluate the diagnostic performance of various biomarkers for PJI and the role of the microbiome in the synovial fluid of patients with prostheses. Patients with suspected knee PJI were enrolled, and their blood and synovial fluid were collected. The cases were classified into the PJI and non-PJI groups. Significant differences between the two groups were observed in the levels of AD (4698 µg/L vs. 296 µg/L, *p* < 0.001) and positivity for LE (62.5% vs. 21.1%, *p* = 0.01). AD had 94.4% sensitivity and 89.5% specificity for diagnosing PJI, whereas LE had 37.5% sensitivity and 100% specificity. Microbiome taxonomic profiling showed high sensitivity. The number of operational taxonomic units and the richness of the microbiome in the synovial fluid were higher in the non-PJI than in the PJI group. AD has shown encouraging results in the Asian population as a diagnostic biomarker for PJI, and LE can be used as a diagnostic adjunct. The bacterial richness of the synovial fluid is likely associated with infections.

## 1. Introduction

Prosthetic joint infection (PJI) is a potentially fatal complication of total joint arthroplasty (TJA). As life expectancy has increased recently, the number of older people who undergo TJA to improve their mobility has also increased. A long duration of prosthesis use, as well as comorbidities associated with advanced age, are linked to periprosthetic infections [1]. The incidence of PJI has been increasing in Korea and globally, resulting in prolonged hospitalization and a substantial increase in medical costs [2,3].

Prompt diagnosis of PJI is important to implementing the most suitable treatment plan; however, the diagnostic criteria are controversial and not definite. Among the most renowned diagnostic criteria for PJI are the 2011 Musculoskeletal Infection Society (MSIS) consensus criteria [4], which were modified during the International Consensus Meeting (ICM) in 2013 [5]. However, these criteria were based on a combination of preoperative and intraoperative markers, and physicians could not diagnose the infection in a timely manner. PJI diagnosis may be missed due to slow-growing organisms or culture-negative infections. Moreover, although the exclusion of persistent infections before replantation in two-stage exchange surgeries for PJI is important to reduce replantation failure, the MSIS criteria showed low sensitivity (0–26%) [6].

α-defensin (AD) is an antimicrobial peptide secreted by human neutrophils as part of the innate immune system defense. It exhibits antimicrobial activities and neutralizes invading pathogens by integrating them into the cell membranes of pathogens [7]. Notably, AD levels in the synovial fluid are elevated in patients with inflammatory joint diseases, including periprosthetic infections [8]. Neutrophils also secrete leukocyte esterase (LE); it has been used to assess urinary tract infections using a dipstick technique [9]. The synovial fluid white blood cell (WBC) count can be estimated through the color changes in LE on the reagent strip, showing a good predictor of PJI [10]. As novel diagnostic biomarkers have demonstrated promising accuracy, new definitions of periprosthetic hip and knee infections have been suggested [11,12]. These latest guidelines suggest the utility of AD using immunoassay or lateral-flow assay as diagnostic tools, but the accuracy of these tests among the Korean population has not been reported. The cut-off value of AD for diagnosis in the Asian population has rarely been assessed. Metagenomic next-generation sequencing is another diagnostic tool for detecting potential pathogens. Evaluating microbial community profiling in the synovial fluid from patients with prostheses could assist in the diagnosis and understanding of PJI. In this study, we aimed to evaluate the diagnostic accuracy of various biomarkers for knee PJI and identify the microbial composition in the synovial fluid of patients with prostheses.

## 2. Materials and Methods

### 2.1. Study Design

In this prospective cohort study, we enrolled 38 patients with suspected PJI after TKA between March 2020 and February 2022 at a 2700-bed tertiary hospital in the Republic of Korea. Blood and synovial fluid samples were collected from the patients who signed the informed consent.

### 2.2. Data and Sample Collection

Demographic data from the patients were collected, including age, sex, underlying conditions, and comorbidities, via electronic medical records. Laboratory results included white blood cell count, erythrocyte sedimentation rate (ESR), and C-reactive protein (CRP) levels. Synovial fluid was aspirated using 18-gauge syringes in a sterile fashion. A synovial sample of 2 mL was immediately delivered to the laboratory to assess WBC and differential counts, protein and glucose levels. In all, 1 mL of synovial fluid samples were processed for aerobic and anaerobic blood culture flasks. Blood cultures were only performed if the clinician suspected sepsis. The samples were cultured for 7 days in general. Periprosthetic tissue pathology was also collected by the clinicians’ decision. Histological analyses were also performed for the tissue.

### 2.3. Definitions

PJI was defined according to the ICM 2013 criteria. The cases that could not be defined according to the ICM criteria were assigned to the non-PJI group. Patients were classified into the PJI group if they met either one major or three (out of five) minor criteria. The major criteria were either two positive periprosthetic cultures with phenotypically identical organisms or a sinus tract communicating with the joint. The minor criteria were elevated CRP and ESR levels, an elevated synovial WBC count, an elevated percentage of synovial polymorphonuclear leukocytes (PMNs), positive histological analysis, and a single positive culture.

### 2.4. Laboratory Methods

The synovial fluid samples were centrifuged to separate cells and other particles, and the resulting supernatant was aliquoted and frozen at −80 °C. The aliquot samples were utilized for the laboratory tests. A total of 0.5 mL of the frozen samples were then transported to SCL healthcare laboratory (Seoul Clinical Laboratories, Yongin, Republic of Korea) for AD detection. AD was detected using a laboratory-based enzyme-linked immunosorbent assay (ELISA) with a Defensin Alpha 1 Neutrophil (DEFa1) kit (SEB705Hu; Cloud-Clone Corp., Houston, TX, USA). The analysis was performed according to the manufacturers’ methods. In summary, it involves binding the target protein to a specific antibody, linking an enzyme to this complex, and then measuring the enzymatic reaction to indicate the concentration of the target protein. Synovial fluid standards were established for consistent assay calibration in the AD immunoassay and the levels were measured in μg/L.

In total, 0.5 mL of the each sample were used for LE detection. This was performed using a urine colorimetric strip (URiSCAN Strip; YD-Diagnostics, Yongin, Republic of Korea). This colorimetric strip test was performed by applying synovial fluid to a reagent test strip, and 2+ or 3+ indicated a positive result. The results were confirmed by three researchers.

Another 0.5 mL of each sample were processed for microbiome taxonomic profiling (MTP). For MTP, PCR amplicons of a phylogenetic marker gene (16S rRNA) were sequenced. Total DNA was extracted using a FastDNA Spin Kit (MP Biomedicals, Seoul, Republic of Korea). PCR amplification was performed using fusion primers targeting the V3–V4 regions of the 16S rRNA gene. Fusion primers of 341F and 805R were constructed in the following order: P5 (P7) graft binding, i5 (i7) index, Nextera consensus, sequencing adaptor, and target region sequence. The amplified DNA fragments were cleansed using the Clean PCR from CleanNA. These purified products, at uniform concentrations, were pooled together and short fragments (non-target products) were removed with CleanPCR (CleanNA). The quality and product size were verified on a Bioanalyzer 2100 (Agilent, Palo Alto, CA, USA) using a DNA 7500 chip. The combined amplicons were pooled and the sequencing was carried out at CJ Bioscience, Inc. (Seoul, Republic of Korea), with an Illumina MiSeq Sequencing system (Illumina, San Diego, CA, USA) as per the guidelines provided by the manufacturer. The processing of raw reads started with a quality check and the filtering out of low-quality (<Q25) reads using Trimmomatic ver. 0.32. The unique reads were extracted, and redundant reads were clustered with the unique reads using the derep_fulllength command of VSEARCH. The EzBioCloud 16S rRNA database [13] was used for a taxonomic assignment using the usearch_global command of VSEARCH, followed by a more precise pairwise alignment. Chimeric reads with <97% similarity were filtered through reference-based chimeric detection using the UCHIME algorithm and the non-chimeric 16S rRNA database from EzBioCloud. After chimeric filtering, reads that were not identified at the species level (<97% similarity) in the EzBioCloud database were compiled, and cluster_fast command2 was used to perform de novo clustering to generate additional operational taxonomic units (OTUs). OTUs with single reads (singletons) were omitted from further analysis. A secondary analysis, which included diversity calculation and biomarker identification, was conducted using the in-house programs of CJ Bioscience, Inc. (Seould, Republic of Korea).

### 2.5. Statistical Analysis

Continuous variables are presented as the median and interquartile range (IQR). Categorical variables are presented as numbers and percentages. Variables and biomarker values were compared between the PJI and non-PJI groups using Mann–Whitney U tests, whereas Fisher’s exact test was used for univariate comparisons of categorical data. The area under the curve (AUC) was obtained using receiver operator curve (ROC) analysis. On the ROC curve, the optimal cut-off value that produced the best combination of sensitivity and specificity was located near the upper-left corner of the curve. We estimated the sensitivity, specificity, positive predictive value (PPV), and negative predictive value (NPV) with 95% confidence intervals (CI) for each diagnostic tool using either Wald or Wilson’s method. In the MTP analysis, the causative organism was determined when its relative abundance in the community was over 10%. The sample microbiota was analyzed using the Chao1 and Shannon indices to evaluate alpha diversity. The Mann–Whitney U test was also used to compare significant differences in the alpha diversity indices between groups. All *p*-values were two-tailed, and a *p*-value < 0.05 was considered statistically significant. Statistical analyses were performed using R Studio (version 4.2.1) and GraphPad Prism 8 software (version 8; Boston, MA, USA).

## 3. Results

**Figure 1:**
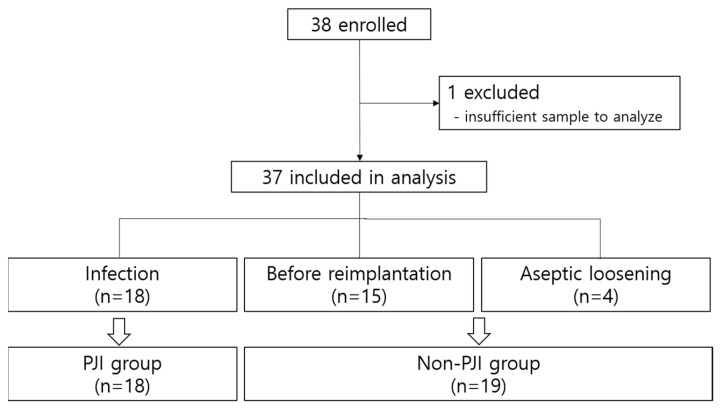
Diagram of the study and classification according to the International Consensus Meeting (ICM) 2013 definition (reference [5]).

Of the 38 enrolled patients, 37 were eligible for this study. One case was excluded because of the scarcity of samples in the operative field. According to the ICM 2013 guidelines, 18 episodes occurred in the PJI group and 19 in the non-PJI group. Definite infections were observed in 48.6% (18/37) of the cases, whereas reimplantation cases accounted for 40.5% (15/37). Four patients (10.5%) had aseptic loosening. There were no cases in which the sinus tract communicated with the joint in our study. The study design is shown in Figure 1.

The demographic and baseline characteristics of the patients with and without PJI are described in Table 1. The age of the patients ranged between 62 to 83 years, and seven patients (18.9%) were male. The median time after TKA was significantly longer in the non-PJI than in the PJI group (97 vs. 32 days, *p* = 0.04). No significant differences in the other variables between the groups were observed.

**Figure 2:**
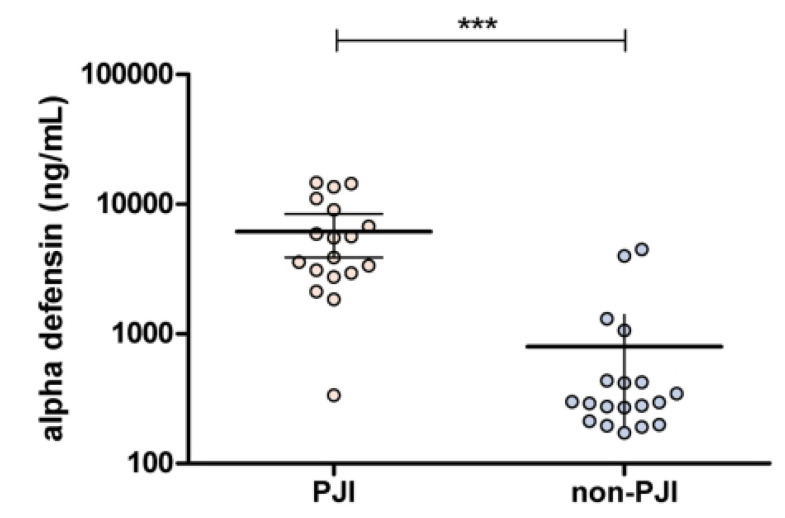
Synovial fluid α-defensin values for PJI and non-PJI cases. PJI, prosthetic joint infection. *** *p* < 0.001.

Synovial fluid AD levels in the two groups were quantitatively measured and compared (Figure 2). The median AD value in the PJI group was significantly higher than that in the non-PJI group AD (4698 vs. 296, *p* < 0.001).

Table 2 shows the laboratory results stratified by group. Compared to the non-PJI group, the PJI group showed significantly higher serum ESR (*p* < 0.001), CRP (*p* = 0.003), and synovial fluid WBC (*p* < 0.001) and PMN levels (*p* < 0.001). In addition, the PJI group showed a significantly different median number of organisms identified by blood culture (*p* < 0.001) and LE levels (*p* = 0.01). In contrast, the number of identified pathogens in the synovial fluid culture was not different between the two groups (*p* = 0.07).

The AUC of AD was 0.93, and the optimal cut-off value for predicting PJI was 1580 μg/L. Table 3 shows the diagnostic performance of the biomarkers. By setting an AD cut-off value of 1580 μg/L, AD showed 94.4% sensitivity (95% CI, 83.9–100%) and 89.5% specificity (95% CI, 75.7–100%). In the blood samples, ESR had moderate sensitivity (70.6%; 95% CI, 49.0–92.2%) and 77.8% specificity (95% CI, 58.6–97.0%). CRP had a moderate sensitivity of 76.5% (95% CI, 56.3–96.6%) and a specificity of 77.8% (95% CI, 58.6–97.0%). Blood culture showed low sensitivity (15.4%; 95% CI, 0–35.0%) and high specificity (100%; 95% CI, 81.6–100%). In the synovial fluid, WBC count and PMN levels had high sensitivity (94.4% and 89.9%, respectively) and specificity (100% and 92.9%, respectively), whereas LE showed a low sensitivity (37.5%; 95% CI, 13.8–61.2%). Synovial fluid culture had relatively low sensitivity (66.7%; 95% CI, 44.9–88.4%) with high specificity (100%; 95% CI, 79.6–100%), whereas MTP indicated high sensitivity (100%; 95% CI, 78.5–100%) but low specificity (55.6%; 95% CI, 23.1–88.0%). LE levels and culture showed 100% positive predictive value, while MTP revealed 100% negative predictive value. Microorganisms isolated from either blood or synovial culture were Staphylococcus aureus (8 samples), Enterococcus faecalis, Staphylococcus epidermidis, Escherichia coli, Candida parapsilosis, and Penicillium species (1 sample each). Microorganisms identified from the synovial samples using the MTP method were *E. coli* (10 samples), *S. aureus* (8 samples), *E. faecalis* (1 sample), and *Bacteroides* spp. (2 samples). Three samples were confirmed as polymicrobial infections. Notably, MTP was superior to either blood or synovial fluid culture.

**Figure 3:**
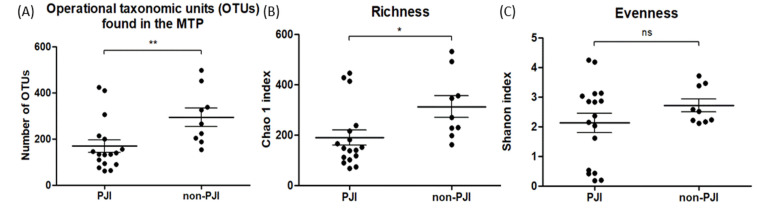
Comparison of species richness and α-diversities in the microbiome taxonomic profile between the PJI and non-PJI groups by (**A**) number of operational taxonomic units (OPUs), (**B**) Chao1, and (**C**) Shannon index. PJI, prosthetic joint infection * *p* < 0.05, ** *p* < 0.01; ns, not significant.

Finally, we compared the number of OTUs and alpha diversity in microbiota samples between the two groups. The number of OTUs identified using MTP was lower in the PJI than in the non-PJI group (133 vs. 265, *p* = 0.006; Figure 3A). The Chao1 index, an indicator of species richness, was lower in the PJI group (148.2 vs. 270.9, *p* = 0.01; Figure 3B) than in the non-PJI group. In contrast, the Shannon index, an indicator of species evenness distribution that considers the number of each species, showed no difference between the groups (2.36 vs. 2.52, *p* = 0.36; Figure 3C).

## 4. Discussion

The diagnosis of PJI after TKA remains challenging because the clinical signs and symptoms are unclear, and elevated levels of a biomarker alone cannot confirm PJI. This study investigated the performance of biomarkers under different conditions and analyzed the microbiome in the synovial fluid. Our results revealed that serum CRP levels and synovial WBC, known biomarkers, were accurate and positive microbial cultures can predict PJI with a high PPV, as reported in other studies [4,5,11,12].

In Korea, AD and LE detection cannot be utilized in clinical practice for the diagnosis of PJI as these tests have not yet received authorization from the Ministry of Food and Drug Safety. Therefore, in the present study, we evaluated these biomarkers in a laboratory setting. In other countries, AD levels can be commercially measured using either an ELISA test developed by CD diagnostics (Claymont, DE, USA) [14] or a point-of-care test using lateral flow tests (Synovasure, Zimmer-Biomet, Warsaw, IN, USA) [15]. The cut-off value for the lateral flow test was set at 5.2 mg/L, based on a previous study in which the evaluation of AD correctly diagnosed 100% of PJI cases, with optimization to the critical value [16]. There was no difference between the AD ELISA and AD lateral flow test results for PJI diagnosis in the pooled cohorts (hip and knee arthroplasty combined), with a sensitivity of 90% vs. 86% (*p* = 0.43) and specificity of 97% vs. 96% (*p* = 0.39) [17]. Moreover, a meta-analysis demonstrated high pooled diagnostic sensitivity and specificity for AD (100% and 96%, respectively) [18]. However, a study on Asian populations reported a relatively low sensitivity of AD (73.7%) compared to that in other populations [19]. In our study, the cut-off value obtained from the ROC curve was 1580 µg/L, which was considerably lower than 5200 µg/L, but it showed comparable good sensitivity and specificity. These results suggest that further research on this biomarker in various ethnicities and races is warranted.

In this study, LE in the synovial fluid had high PPV and specificity but low sensitivity. In contrast, a previous review showed that the sensitivity of the strip test for the diagnosis of PJI was 85.7% [18]. The reason for this discrepancy is that the strip test can be influenced by the subjective judgment of the tester and other confounding factors, including bloody samples. In addition, freeze-thawing of the samples may have influenced the result. However, this diagnostic tool can be used in the field because of its convenience, low cost, and rapid turnaround time [20]. Therefore, positive findings in the clinical field can still be considered reliable for diagnosis and may lead to additional treatment interventions.

Metagenomic next-generation sequencing showed high accuracy for diagnosing PJI in a meta-analysis [21]. In our study, among the 18 PJI episodes, 13 were culture-positive cases (11, synovial culture only; 1, blood culture only; 1, both samples); the pathogens identified through culture were also detected via MTP in 8 cases. However, two pathogens were confirmed using the MTP method in culture-negative samples; other pathogens were identified in culture-positive samples. Other studies showed metagenomic next-generation sequencing can identify a wide range of pathogens, although the presence of human and contaminant microbial DNA from reagents is challenging [22,23]. The utility of 16S rRNA gene-based targeted metagenomic sequencing was comparable to that of metagenomic next-generation sequencing [24]. In our study, potential pathogens that were missed by microbial culture were also detected through MTP. MTP in the non-PJI group indicated a higher microbial richness (using the Chao1 index) than in the PJI group; the OTU level was also higher in the non-PJI group. OTUs are often used to infer functional traits because they represent community members. Therefore, lower microbial richness in the synovial fluid was associated with an increased likelihood of periprosthetic infections. A causal relationship between PJI and microbiome diversity was not identified in this study. The existence of bacterial nucleic acids in the normal synovial fluid has been confirmed; however, the underlying mechanism remains unclear [25]. Prosthesis in a joint can generate an environment in which bacteria can form biofilms, providing a beneficial survival system for some community members. When biofilms release planktonic forms of bacteria from the surfaces upon maturation [26], they may persistently disrupt the diversity and composition of microbial communities. Hence, the role of the microbiome in prosthetic joints should be further investigated.

This study had some limitations. First, accuracy tests were performed using small samples collected from knee periprosthetic joint aspiration. However, because these samples were prospectively collected and the PJI diagnosis was evaluated retrospectively, our findings could have resulted in reduced bias. In addition, all immunoassays were performed in one laboratory and thus require further validation. As the microbiomes of the periprosthetic synovial fluid were evaluated by profiling the V3/V4 regions of bacterial 16S rRNA genes, the presence of other microorganisms, such as fungi, was not investigated. This limitation could be overcome by combining microbial culture or a fungal sequencing target. Lastly, the AD and LE tests were performed on the samples after undergoing freeze-thawing, which could not determine the treatment plan. It might have affected the performance. Therefore, to confirm our findings, large-scale studies involving multicenter hospitals with a wide range of PJI cases among Asians should be conducted for reliable results.

## 5. Conclusions

In conclusion, we confirmed that for detecting PJI synovial fluid AD is a potent biomarker, compared to other biomarkers in the Asian population. Synovial fluid LE levels can also be used as a diagnostic adjunct. Moreover, we also showed that the bacterial richness of the synovial fluid is likely associated with periprosthetic infections.

## Figures and Tables

**Table 1:** Baseline characteristics of patients with and without PJI.

Variables	PJI (*n* = 18)	Non-PJI (*n* = 19)	*p*-Value
Male	5 (10.5)	2 (27.8)	0.23
Age, years, median [IQR]	73 [70–79]	78 [71–81]	0.63
BMI, kg/m^2^, mean ± SD	26.5 ± 3.7	26.9 ± 4.5	0.77
Underlying disease			
Hypertension	14 (77.8)	11 (57.9)	0.30
Diabetes mellitus	6 (33.3)	8 (42.1)	0.74
CAOD	3 (16.7)	0 (0)	0.11
CVA	2 (11.1)	5 (26.3)	0.40
Time after TKA, months, median [IQR]	32 [21–49]	97 [41.0–184]	0.04
Previous antibiotics, yes	8 (50)	5 (27.8)	0.29

Data are presented as numbers (%) for categorical variables. PJI, prosthetic joint infection; IQR, interquartile range; BMI, body mass index; SD, standard deviation; CAOD, coronary artery occlusive disease; CVA, cerebrovascular accident; TKA, total knee arthroplasty.

**Table 2:** Laboratory results associated with PJI.

Variables	PJI	Non-PJI	*p*-Value
*Blood*			
ESR, mm/h, median [IQR]	62 [29.5–114.0]	18.5 [9.3–27.8]	<0.001
CRP, mg/dL, median [IQR]	97 [10.4–120]	1.6 [1.0–4.9]	0.003
Culture ^1^	12 (66.7)	0	<0.001
*Synovial fluid*			
Alpha defensin, median [IQR]	4698 [2986–8466]	296 [241–429]	<0.001
WBC count, /μL, median [IQR]	30250 [20818–60300]	550 [240–810]	<0.001
PMN (%), median [IQR]	91.5 [85.3–94.0]	15.5 [3.0–54.8]	<0.001
LE, positive (%)	10 (62.5)	4 (21.1)	0.01
Culture ^1^ (%)	3 (23.1)	0	0.07

Data are presented as numbers (%) for categorical variables. ^1^ Positive culture of the causative organism. ESR, erythrocyte sedimentation rate; CRP, C-reactive protein; WBC, white blood cell; PMN, polymorphonuclear leukocyte; LE, leukocyte esterase.

**Table 3:** Sensitivity, specificity, positive predictive value, and negative predictive value of the blood and synovial tests.

Tests	Sensitivity	Specificity	Positive Predictive Value	Negative Predictive Value
*Blood*				
ESR	70.6 (48.99–92.2)	77.8 (58.6–97.0)	75 (53.8–96.2)	73.7 (53.9–93.5)
CRP	76.5 (56.3–96.6)	77.8 (58.6–97.0)	76.5 (56.3–96.6)	77.8 (58.6–97.0)
Culture ^1^	15.4 (0–35.0)	100 (81.6–100)	100 (34.2–100)	63.0 (44.7–81.2)
*Synovial fluid*				
Alpha defensin	94.4 (83.9–100)	89.5 (75.7–100)	89.4 (75.7–100)	94.4 (83.9–100)
WBC	94.4 (83.9–100)	100 (79.6–100)	100 (90.2–100)	93.8 (81.9–100)
PMN (%)	88.9 (74.4–100)	92.9 (79.4–100)	94.1 (83.0–100)	86.7 (69.5–100)
LE, positive	37.5 (13.8–61.2)	100 (83.2–100)	100 (61.0–100)	65.5 (48.2–82.8)
Culture ^1^	66.7 (44.9–88.4)	100 (79.6–100)	100 (75.8–84.1)	71.4 (52.1–90.8)
MTP ^2^	100 (78.5–100)	55.6 (23.1–88.0)	77.8 (58.6–97.0)	100 (56.6–100)

Values are expressed as percentages (95% confidence interval) using either Wald or Wilson’s method. Tests were assessed according to the International Consensus Meeting (ICM) 2013 definition adapted from Parvizi and Gehrke (reference [5]) unless other captions were described. ^1^ Positive culture of the causative organism. ^2^ Detection of the causative organism by sequencing PCR amplicons of a phylogenetic marker gene (16S rRNA gene). ESR, erythrocyte sedimentation rate; CRP, C-reactive protein; WBC, white blood cell; PMN, polymorphonuclear leukocyte; LE, leukocyte esterase; MTP, microbiome taxonomic profiling.

## Data Availability

The datasets used and/or analyzed during the current study are available from the corresponding author upon reasonable request.
